# The Differential Effects of Public and Private Active Social Media Use on Adolescents’ Well-Being: Insights from a Cross-Sectional and Experimental Study

**DOI:** 10.3390/bs16091543

**Published:** 2026-09-01

**Authors:** Jing Qiao, Ming Ji

**Affiliations:** 1 Teacher Development College, Shaanxi Normal University, Xi’an 710062, China; 2 Affiliated High School of Guizhou Normal University, Guiyang 550025, China; 3 School of Psychology, Shaanxi Normal University, Xi’an 710119, China

**Keywords:** public active social media use, private active social media use, perceived online social support, well-being

## Abstract

While existing research on active social media use and well-being has yielded inconsistent findings, this study introduces a refined typology distinguishing between public (e.g., posting broadly on social networks) and private (e.g., one-on-one messaging) active use to clarify these effects. It further examines the moderating role of perceived online social support. Study 1 (*N* = 689) used a cross-sectional survey design to analyze the relationships among public versus private active social media use, perceived online social support, and well-being in a population of Chinese adolescents. Building upon these findings, Study 2 (*N* = 158) used a scenario-based experiment to further validate the findings of Study 1. Both studies found that public active social media use had a stronger effect on well-being than private active social media use. Perceived online social support moderated the relationship between public active social media use and well-being. Specifically, under the public active social media use condition, participants in the low online social support group were less likely to report well-being than those in the high online social support group. However, under the private active social media use condition, there was no difference between the high and low support groups. This study provides new insights into the relationship between active social media use and well-being. However, the moderation effect was modest in magnitude, and Study 2 relied on a single-item well-being measure; the directional conclusions should therefore be interpreted with appropriate caution.

## 1. Introduction

Well-being plays a crucial role in individuals’ overall mental and physical health (Orben & Przybylski, 2019). It is closely intertwined with multiple aspects of daily life, including personal relationships, work satisfaction, and financial stability. Many factors can influence a person’s well-being, such as their physical activity, environmental conditions, and individual personality traits (Zhou et al., 2024). A recent study has shown that nearly 4.8 billion people have used at least one social media platform (Petrosyan, 2023). As social media becomes increasingly embedded in everyday life, its use has been shown to have both positive and negative effects on individuals’ well-being (Nguyen et al., 2025). A primary area of scholarly interest lies in active social media use (ASMU), which involves intentional interactions such as posting, commenting, and engaging directly with other users on social media (Valkenburg et al., 2022). Empirical studies on the relationship between ASMU and well-being, however, present a complex and often inconsistent picture. While some studies have linked ASMU to positive psychological outcomes (e.g., Hancock et al., 2019; Lian et al., 2020), others have found adverse effects such as an elevated risk of depression (e.g., Frison & Eggermont, 2020). However, to date, few studies have examined why ASMU produces such divergent results. This inconsistency underscores the need for further exploration of the nuanced role of active social media use in shaping individual well-being.

We propose that these inconsistent findings may stem from the lack of differentiation between public and private ASMU. The Uses and Gratifications Theory implies that the impact of social media on well-being depends largely on how and why individuals engage with these platforms (Panova & Carbonell, 2018). Prior studies have found that active social media use can be divided into two categories: public and private ASMU (Valkenburg et al., 2022). Public ASMU consists of behaviors that involve visible, interactive activities on social media platforms (i.e., posting status updates), while private ASMU involves more personal, one-on-one interactions conducted within a private or semi-private space on social media (i.e., sending direct messages) (Verduyn et al., 2015). In addition, the present study seeks to examine moderating mechanisms that may account for the divergent effects of public and private ASMU on psychological well-being. Compensatory Use Theory implies that perceived online social support may serve as a functional response to negative affective states (Kardefelt-Winther, 2014). Thus, perceived support may moderate the effect of different types of ASMU on well-being.

Based on this, the research questions of this study are as follows. (1) Do different types of social media use have different effects on well-being? (2) Does perceived online social support moderate the relationships linking the different types of ASMU to well-being? To empirically answer these questions, this study uses a mixed-methods approach, incorporating both survey-based assessments and a situational experimental paradigm. This approach was chosen as a way to isolate the distinct effects of public versus private ASMU, while accounting for individual differences and external factors. This permits a nuanced examination of how variations in perceived support may moderate the psychological outcomes associated with different types of ASMU.

### 1.1. The Relationship Between ASMU and Well-Being

Researchers are increasingly recognizing that ASMU is not merely a form of communication, but carries potentially highly significant implications for individuals’ psychological health and subjective well-being as well. ASMU has become an integral part of daily life, but its impact on well-being has been suggested to vary depending on whether the use is public or private (Valkenburg et al., 2022). Public active social media use, in which individuals post content, share experiences, and engage with a broad audience, has been shown to have the potential to promote well-being, particularly in terms of social validation and community belonging (Thorisdottir et al., 2019). When people share positive experiences and achievements publicly, they often receive supportive comments, likes, and shares, all of which can boost their self-esteem and enhance their sense of social connectedness (Winstone et al., 2021). This public affirmation can be especially rewarding, as it provides a sense of recognition and visibility that the individual may find lacking in offline contexts. Moreover, public engagement on social media platforms allows individuals to establish a personal brand or identity that can be associated with success, creativity, and meaningful contributions (Harris & Rae, 2011). These positive feedback loops can foster a greater sense of purpose and happiness, especially when the individual feels supported by a larger community of followers or friends (Thorisdottir et al., 2019). However, this benefit is not uniform: by broadcasting personal content to broad audiences, it can also expose users to upward social comparison, impression-management pressure, and fear of missing out, which have been linked to diminished self-evaluation and lower affective well-being (Verduyn et al., 2020; Vogel et al., 2014).

By contrast, although private active social media use can facilitate intimate connections with close friends or family through direct messaging or private groups, it does not necessarily guarantee the same positive outcomes (Gruzd & Hernández-García, 2018). Private interactions are often more constrained and may not provide the same level of social validation as public sharing. Furthermore, the nature of private social media use can sometimes lead to negative psychological effects. For instance, individuals may rely too heavily on a small, familiar group of people for emotional support, which can lead to feelings of dependency or reduced autonomy (O’Day & Heimberg, 2021; Hartanto et al., 2025). Private use can also exacerbate feelings of loneliness or social isolation, particularly if individuals experience a lack of engagement or their online interactions fail to meet their emotional needs (Valkenburg et al., 2022). Moreover, the intimate nature of private communication may encourage individuals to compare their own lives to the curated, idealized versions of the lives of others that they encounter in these spaces (Brandenberg et al., 2019), which can reduce their overall happiness. Nevertheless, this detriment is not uniform: through directed one-to-one communication, it can also foster intimate relationship maintenance, emotional disclosure, and perceived social support (Burke & Kraut, 2016; Oh et al., 2014).

We thus propose as follows:

**Hypothesis** **H1.** *Public active social media use has a positive effect on well-being, while private active social media use has a negative effect on well-being.*

### 1.2. The Relationship Between Perceived Online Social Support and Well-Being

Online social support is the sense of recognition and belonging that individuals obtain through emotional, informational, and material exchanges in virtual interactions, in which they feel understood and respected by others (Frison & Eggermont, 2016). The nature of digital communication makes online social support inherently different from traditional face-to-face support, as online interactions tend to lack concealability and are often marked by a high degree of self-presentation (Sun et al., 2024). In face-to-face interactions, the nuances of body language, vocal tone, and physical presence can significantly shape the experience of support in ways that make it more personal and emotionally direct. By contrast, online interactions tend to be more filtered, with individuals often curating their self-presentation to create specific public personas. This dynamic, coupled with the relative anonymity of digital platforms, can influence the perceived sincerity and emotional depth of the support exchanged online (Mondal et al., 2020).

Perceived online social support, specifically, refers to an individual’s subjective appraisal and belief in the availability, adequacy, and effectiveness of such support within their social ecosystem. This distinction is crucial as it emphasizes the personal interpretation of supportive resources rather than their objective presence. Research has consistently shown that individuals’ perceptions of the online support they receive from their social network play a crucial role in shaping their emotional and psychological well-being (Cohen & Wills, 1985). Specifically, online social support can mitigate the negative effects of stressors by offering individuals a sense of reassurance, empathy, and understanding in challenging times (Trepte et al., 2015; Nabi et al., 2013). Empirical research has shown that higher levels of perceived online social support are associated with reduced loneliness and depression, as well as increased life satisfaction and emotional well-being (Utz & Breuer, 2017). Social support obtained from social media can thus be gratifying and reinforce the behavior of seeking out social interactions online, potentially leading to problematic levels of internet engagement (Cheng et al., 2019, Wei et al., 2024). We thus propose as follows:

**Hypothesis** **H2.** *Perceived online social support is positively associated with well-being.*

In addition, the degree to which individuals perceive social media as a source of support can potentially influence the impact of ASMU, whether public or private, on their well-being. When individuals perceive social media interactions as being supportive and emotionally meaningful, they are more likely to experience the psychological benefits of public ASMU (Cobo-Rendón et al., 2020). Conversely, if these interactions are viewed as being superficial or lacking in support, the positive effects of public ASMU may be weakened. This suggests that the impact of ASMU may depend on the individual’s perception of the supportiveness of their social network. Specifically, in contexts in which perceived online social support is low, public ASMU may fail to yield positive outcomes and could instead potentially exacerbate negative emotional experiences. In contrast, in contexts where perceived support is high, individuals are more likely to successfully fulfill their social or emotional needs through public ASMU, leading to greater levels of well-being (Godard & Holtzman, 2024).

We thus propose the following hypothesis:

**Hypothesis** **H3.** *Perceived online social support moderates the effect of public ASMU on well-being. Specifically, when perceived online social support is low, public ASMU does not significantly predict well-being. However, when perceived online social support is high, public ASMU is positively associated with well-being.*

Similarly, the effect of private ASMU on well-being also appears to depend on the level of perceived social support. When perceived support is low, private ASMU may not generate meaningful psychological benefits and may even intensify negative emotions (Frison & Eggermont, 2015). Conversely, when perceived support is high, private ASMU is more likely to satisfy individuals’ social and emotional needs, thereby enhancing their overall well-being (Godard & Holtzman, 2024). We thus propose as follows:

**Hypothesis** **H4.** *Perceived online social support moderates the effect of private ASMU on well-being. Specifically, when perceived online social support is low, private ASMU does not significantly predict well-being. However, when perceived online social support is high, private ASMU is positively associated with well-being.*

### 1.3. The Current Study

**Figure 1:**
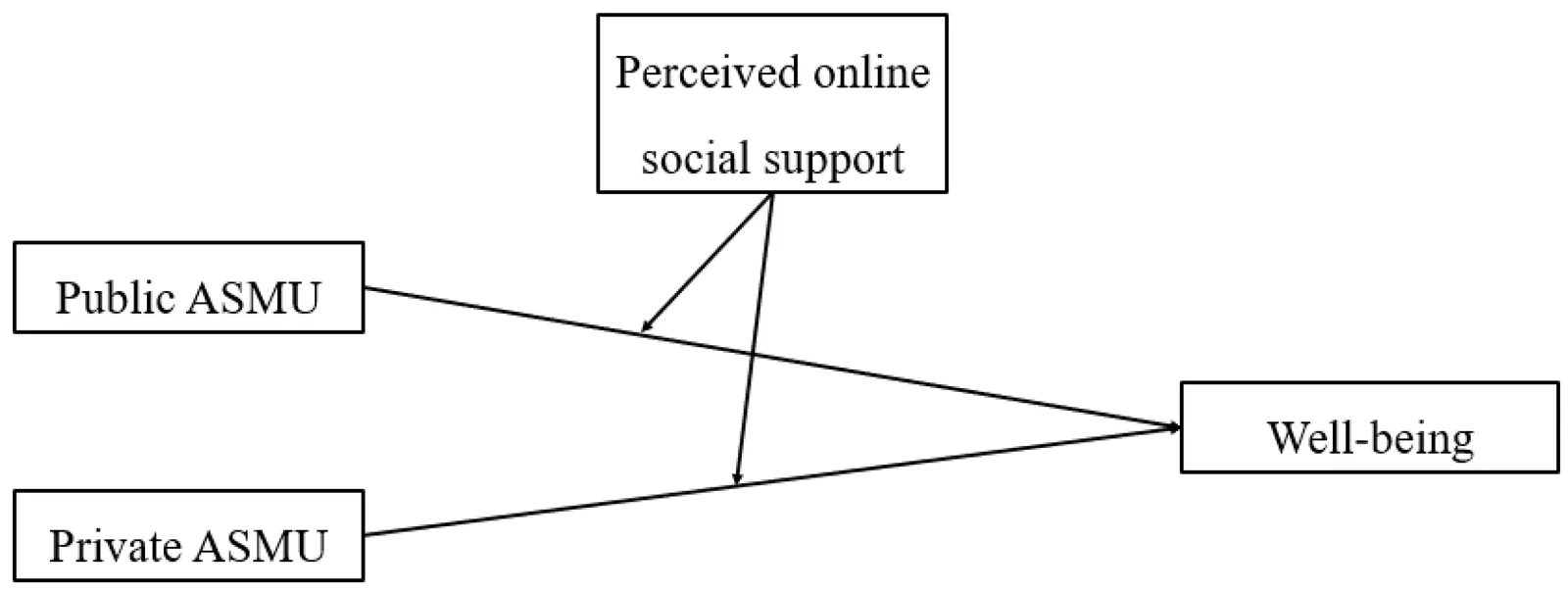
The hypothesized model.

To test our hypotheses, we conducted two complementary studies. Study 1 used a questionnaire method to gather real-world evidence regarding the relationships among public and private active social media use, perceived online social support, and psychological well-being. A questionnaire was administered to a large sample of social media users, which included measures of their frequency and type of ASMU (i.e., public vs. private), their perception of the support they have received from their social media interactions, and their overall psychological well-being. Study 2 sought to experimentally manipulate the type of social media interaction (i.e., public vs. private ASMU) and the perception of support to establish causal relationships. In this experiment, participants were randomly assigned to engage in either public or private social media interactions, with the perception of support being manipulated through varying types of feedback. Figure 1 depicts the hypothesized model, including the hypotheses and research questions.

## 2. Study 1: A Cross-Sectional Analysis of Public and Private ASMU in Relation to Adolescent Well-Being

### 2.1. Participants

Study 1 was approved by the relevant research ethics committee of the authors’ university. A total of 703 participants were recruited from diverse educational institutions in China through an online survey tool (Qualtrics). The final sample consisted of 689 questionnaires, after exclusion of 14 participants who failed to complete all the sessions. The final sample comprised 165 males (23.95%) and 524 females (76.05%) with a mean age of 16.97 years (*SD* = 1.34 years), ranging from 14 to 19 years. In terms of grade level, there were 197 grade 10 students (28.59%), 228 grade 11 students (33.09%), and 264 grade 12 students (38.32%). In the Chinese educational system, grades 10–12 generally correspond to ages 15–18, though individual variation of approximately ±1 year may occur due to differences in school entry age—a pattern broadly consistent with U.S. secondary schools, where grades 10, 11, and 12 enroll roughly comparable age cohorts.

Participants were included if they possessed at least one active social media account with a usage record in the past month and provided informed consent (guardian consent was required for minors). Exclusion criteria comprised a diagnosis of a mental disorder or receipt of psychotherapy within the past 6 months, as well as social media usage frequency below once per month (extreme low users). All exclusion criteria were assessed through participants’ self-report, as no clinical diagnostic records or external verification were available to the research team. Both studies employed a convenience sampling approach by posting advertisements on social media platforms to recruit participants, who received a monetary reward upon completion of the study.

In the present study, adolescent participants reported on their active use of WeChat 8.0.76, the predominant social media platform among Chinese adolescents. Public active social media use was operationalized as behaviors broadcast to a broad audience—posting status updates or photos on WeChat Moments, and liking or commenting on others’ publicly visible content. Private active social media use comprised behaviors confined to dyadic exchanges—one-on-one messaging and voice messages on WeChat. Within WeChat, Moments serves as the public, broadcast-oriented space, whereas one-on-one chat represents the private, dyadic space; this functional distinction underpins our classification of public versus private active use. The same WeChat-based operationalization was adopted in Study 2, where the experimental scenarios were presented as simulated WeChat interfaces (WeChat Moments for the public condition and a WeChat one-on-one conversation for the private condition).

### 2.2. Procedure

The study was conducted as an online cross-sectional survey. Participants accessed the questionnaire via Qualtrics and completed it at a time and location of their convenience (e.g., a school computer laboratory or a personal device). The questionnaire was administered in a fixed order for all participants: demographic questions (age, grade, gender), followed by the active social media use scales, the perceived online social support scale, and the well-being scale. Informed consent was obtained before the questionnaire commenced, and participants were debriefed upon completion.

### 2.3. Measures

#### 2.3.1. Public and Private ASMU Scale

Public ASMU was measured using two items adapted from prior studies (Frison & Eggermont, 2020), specifically “How often do you post a message on your social network,” and “How often do you post a photo on your social network” (*r* = 0.67). Private ASMU was similarly measured using two items adapted from prior studies (Frison & Eggermont, 2020), specifically “How often do you send someone a personal message on your social network,” and “How often do you chat with someone on your social network” (The Pearson *r* between the two items was = 0.72). Participants rated each item on a 5-point scale from 1 = *never* to 5 = *frequently*. For each dimension, the two item scores were averaged to yield a composite score ranging from 1 to 5, with higher scores indicating more frequent engagement in that type of active social media use.

#### 2.3.2. Perceived Online Social Support Scale

To measure respondents’ perceptions of online social support, a four-item measure was developed based on the family subscale of the Multidimensional Scale of Perceived Social Support (Zimet et al., 1988). Respondents evaluated four items using a five-point scale from 1 = *strongly disagree* to 5 = *strongly agree*. Responses to the four items were averaged to create a composite score ranging from 1 to 5, with higher scores indicating greater perceived online social support. Each item started with “When you are feeling down or in a difficult situation…”, followed by: (1) “I can find help on the social network”, (2) “I can find the emotional help and support that I need on the social network”, (3) “I can talk with someone on the social network about my problems”, and (4) “I can find someone on the social network that helps me make decisions.” In the current study, the internal consistency of this scale was high (ɑ = 0.92). Because this measure was adapted from the family subscale of the MSPSS, which has an established unidimensional structure (Zimet et al., 1988), and the adaptation only recontextualized the source of support from family members to online social networks without altering the theorized dimensionality, CFA rather than EFA was conducted to examine whether the established one-factor structure was supported in the present adolescent sample. The results of confirmatory factor analysis (CFA) in this study indicate a good fit with the data: χ^2^/df = 3.49, comparative fit index (CFI) = 0.97, non-normed fit index (NNFI) = 0.97, goodness-of-fit index (GFI) = 0.98, and root mean square error of approximation (RMSEA) = 0.06. Each of the scale items was significantly loaded on its intended underlying factor. Adopting the conventional a priori criterion of standardized factor loadings ≥0.40 recommended by Hair et al. (2019), all four items were retained; the actual standardized loadings all exceeded 0.69.

#### 2.3.3. Well-Being Scale

Well-being was assessed by averaging eight items from the Psychological Well-Being Scale (PWB; Diener et al., 2009). A sample item is, “My social relationships are supportive and rewarding”; all items were ranked on a seven-point Likert scale from 1 = *strongly disagree* to 7 = *strongly agree*. Responses to the items were averaged to create a composite score ranging from 1 to 7, with higher scores indicating higher levels of psychological well-being. In this study, the internal consistency of this scale was high (ɑ = 0.94). The results of confirmatory factor analysis (CFA) in this study indicate a good fit with the data: χ^2^/df = 4.99, comparative fit index (CFI) = 0.95, non-normed fit index (NNFI) = 0.95, goodness-of-fit index (GFI) = 0.94, and root mean square error of approximation (RMSEA) = 0.07. Each of the eight scale items was significantly loaded on its intended underlying factor. Adopting the conventional a priori criterion of standardized factor loadings ≥0.40 (Hair et al., 2019), all items were retained; the actual standardized loadings all exceeded 0.69.

### 2.4. Statistical Analyses

Moderated mediation analysis was conducted using the PROCESS macro developed by Hayes (2017) in SPSS. Mediation models were constructed with public and private ASMU as the independent variable, well-being as the dependent variable, and perceived online social support as the moderating variable. Gender was covaried in each model. Parameters were estimated using the percentile bootstrapping method with replacement (5000 samples). Effects were considered significant if the 95% confidence interval (CI) estimates excluded zero. Analyses were carried out using SPSS Version 21.0 and PROCESS3.0. All analyses were performed using SPSS (Version 25; IBM Corp., Armonk, NY, USA).

### 2.5. Results

#### 2.5.1. Common Method Variance Analysis

Harman’s one-factor test was used to assess potential common method bias. The results indicated seven factors extracted; the first common factor explained 20.25% of the total variance. As this was less than 40% (Podsakoff et al., 2003), this result indicates that common method variance was not a serious problem in this study.

#### 2.5.2. Preliminary Analyses

Descriptive statistics were computed for each measurement. Table 1 presents the means, standard deviations, and Pearson product-moment correlations of the main study variables. As expected, public ASMU and private ASMU were both positively associated with perceived online social support. Furthermore, public ASMU was positively associated with well-being. However, private ASMU was not significantly associated with well-being (*p* > 0.05).

Before conducting the moderated mediation analyses, we examined the assumptions underlying multiple regression (Tabachnick & Fidell, 2019). Normality of the dependent variable was assessed via skewness and kurtosis (all absolute values < 3.00; Kline, 2016); multicollinearity among predictors was evaluated using variance inflation factors (all VIFs < 5.00; Hair et al., 2019); homoscedasticity was verified through [Breusch–Pagan test, *p* > 0.05/examination of residual plots]; linearity was confirmed by examining partial regression plots; and the independence of observations was ensured by the cross-sectional, independently sampled design (no repeated measures or clustered sampling). All assumptions were satisfactorily met.

#### 2.5.3. Hypothesis Testing

**Figure 2:**
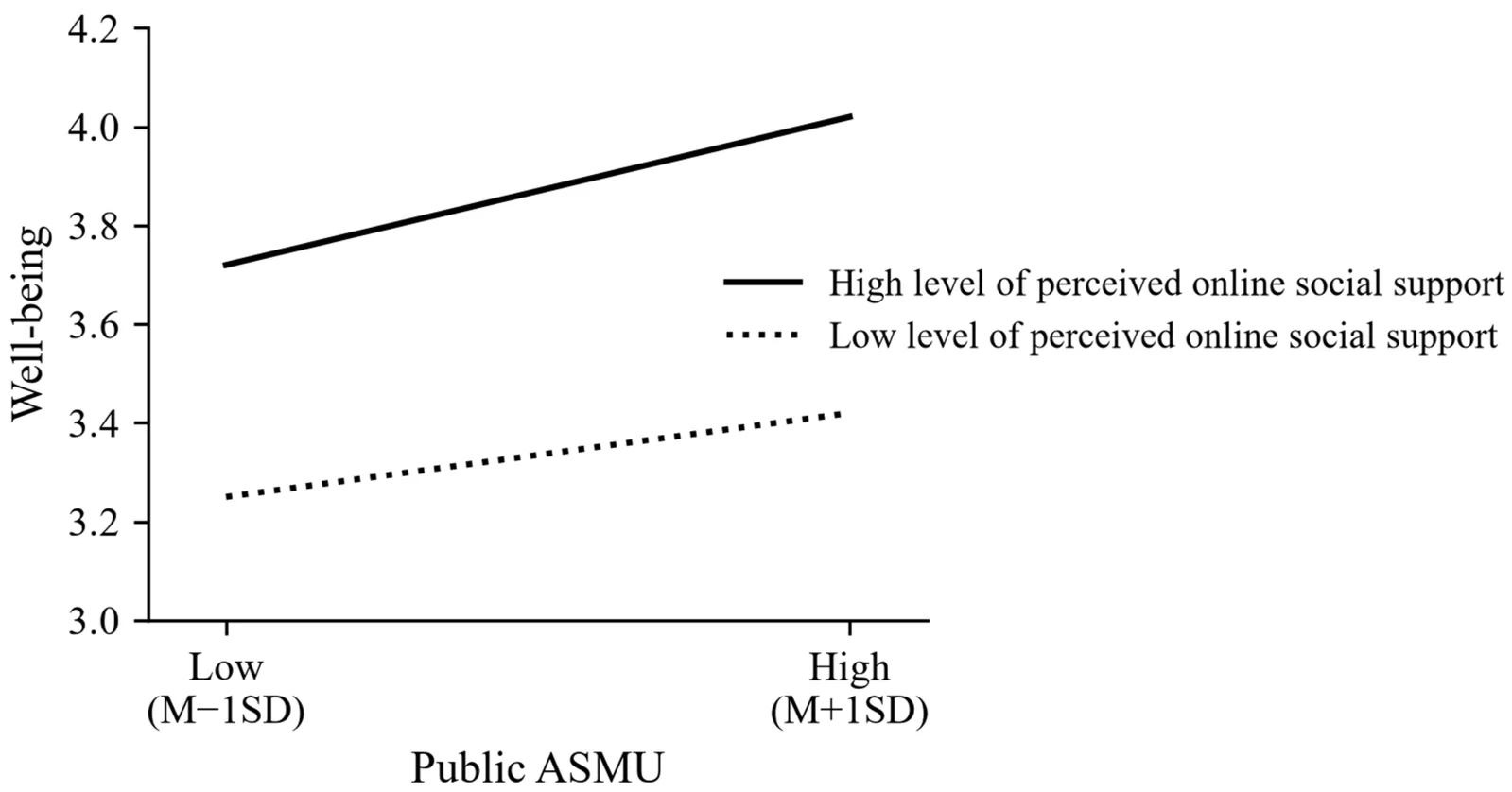
The association between public ASMU and well-being at high and low levels of perceived online social support.

Table 2 presents the results of the moderation analyses. First, public ASMU was positively associated with well-being (β = 0.15, *p* < 0.01), but private ASMU was not significantly associated with well-being (β = −0.01, *p* > 0.05). Second, perceived online social support was significantly positively associated with well-being (β = 0.21, *p* < 0.01). Third, perceived online social support moderated the relationship between public ASMU and well-being (β = 0.06, *p* < 0.05). A simple slope test was conducted for a low level of the moderator (M − 1 SD) and a high level of the moderator (M + 1 SD) to better clarify the moderation effect (Hayes, 2017). The results (see Figure 2) indicate that public ASMU was more strongly associated with well-being in adolescents with high levels of perceived online social support (β = 0.18, *p* < 0.01) but less strongly associated in those with low levels of perceived online social support (β = 0.11, *p* < 0.01). The moderation effect was small in magnitude (*f*^2^ = 0.05), indicating that perceived online social support accounts for a limited proportion of variance in the public ASMU–well-being link. Although the simple-slope pattern was statistically significant (β = 0.18 at high support vs. β = 0.11 at low support), the effect size is small; this moderation should therefore be interpreted cautiously and replicated with larger, more sensitive samples before strong conclusions are drawn.

Meanwhile, private ASMU was not significantly associated with well-being (β = 0.06, *p* > 0.05). Perceived online social support did not moderate the relationship between private ASMU and well-being (β = −0.01, *p* > 0.05).

### 2.6. Discussion

This study, based on questionnaire survey data, found that public ASMU had a significant positive effect on adolescents’ well-being, whereas private ASMU did not exhibit a significant impact. This discrepancy may be due to differences in the frequency of engagement and nature of public and private ASMU. Prior research suggests that individuals tend to engage in private ASMU (e.g., direct messaging) more frequently than public ASMU (e.g., posting status updates) (Valkenburg et al., 2022). Moreover, private interactions often involve routine, dyadic communications that may lack novelty and emotional salience, thereby exerting a weaker influence on users’ psychological outcomes (Verduyn et al., 2022). By contrast, public ASMU is more likely to elicit broader social visibility and feedback, which can enhance feelings of social connectedness and validation, thereby improving well-being.

Further analysis indicated that the effect of public ASMU on well-being was moderated by perceived online support. Specifically, when perceived support was high, the beneficial impact of public ASMU on well-being was more pronounced; conversely, when perceived support was low, this positive effect was substantially weakened. These findings underscore the importance of the social support derived from content shared on social network platforms. Many individuals post content on social media in hopes of garnering attention, and the absence of such support can result in adverse effects on their well-being, as can exposure to negative feedback (Lian et al., 2020). When adolescents perceive greater online social support, it fosters more positive life attitudes, enhances their sense of confidence and security, and strengthens their willingness to help others, all of which can collectively contribute to elevated levels of well-being (Xu & Han, 2024). Upon further reflection on the pattern shown in Figure 2, the figure revealed that at low public ASMU, adolescents reporting high perceived online social support exhibit higher well-being than adolescents with low perceived support who report high public ASMU. Although the pattern shown in Figure 2 may initially appear counterintuitive, it suggests that perceived online social support may be more important for adolescent well-being than the frequency of public ASMU itself. Perceived online social support provides adolescents with a sense of belonging, emotional security, and social validation, thereby promoting well-being (Cohen & Wills, 1985; Xu & Han, 2024). In contrast, the potential benefits of public ASMU may depend on whether such engagement elicits supportive responses. Without supportive feedback, frequent public posting may provide limited benefits and may even expose adolescents to unfavorable social comparison or negative feedback (Lian et al., 2020). Thus, the pattern in Figure 2 suggests that cultivating a supportive online environment may be more important for adolescent well-being than simply increasing the frequency of public ASMU.

It is also important to note a demographic characteristic of Study 1 that bears on the interpretation and generalizability of the findings. The sample was substantially female-skewed, with 524 girls (76.05%) and 165 boys (23.95%) among the 689 participants (*M*_age_ = 16.97 years, *SD* = 1.34; range = 14–19 years). Given this gender imbalance, gender was included as a covariate in all moderated-mediation models to statistically account for potential effects associated with gender. Nevertheless, the strong female predominance may limit the generalizability of the findings to more gender-balanced adolescent populations. Future research should replicate these findings using larger samples with more balanced gender distributions or stratified sampling.

In contrast, private ASMU was found to be neither directly associated with well-being nor significantly moderated by perceived online social support. This may indicate that private ASMU, while instrumental for maintaining close interpersonal relationships, is not a robust source of emotionally salient or socially rewarding experiences that can influence broader indicators of well-being. It should be noted that H1 additionally predicted a negative effect of private ASMU on well-being; the observed association was non-significant rather than negative, which constitutes a partial disconfirmation of H1. In other words, whereas the positive effect of public ASMU was supported, the predicted negative effect of private ASMU was not borne out.

It is worth noting that these findings are derived from a cross-sectional design, which limits the ability to draw causal inferences. To address this limitation and further validate the observed moderation effect, Study 2 was devised, using a scenario-based experimental design. This approach allows for more precise manipulation of perceived online support and ASMU type and thus can provide initial causal evidence regarding the directionality of the observed effects, although the moderation effect was modest in magnitude and subject to the ecological constraints noted below.

## 3. Study 2: An Experimental Test of the Impact of Public vs. Private ASMU on Adolescents’ Well-Being

### 3.1. Participants

Study 2 was approved by the relevant research ethics committee of the authors’ university. Written informed consent was obtained from all participants, with parental/guardian consent required for minors. The study was approved by the Institutional Review Board of Shaanxi Normal University (protocol code HR2024-7-17). Prior to the experiment, a priori sample size calculation was conducted using G*power 3.1.9.2 (Faul et al., 2007). Assuming an alpha (α) of 0.05, a power of 0.8, and four groups, the total sample size needed to detect a medium effect size (f^2^ = 0.15) was determined to be 128 (ANCOVA, fixed effects, main effects, and interactions). The data were collected online using Wen Juan Xing, a professional online survey platform comparable to MTurk. A total of 158 adolescents, comprising 96 females and 62 males, were recruited. Their mean age was 17.09 ± 1.03 years (ages ranging from 15 to 19 years). The participants were randomly assigned to four conditions in a 2 (public ASMU vs. private ASMU) × 2 (high online social support vs. low online social support) between-subjects design. The study was carried out in accordance with the guidelines for human subjects research.

Participants were randomly assigned to one of four experimental conditions: private ASMU–low perceived online social support, private ASMU–high perceived online social support, public ASMU–low perceived online social support, and public ASMU–high perceived online social support. The private ASMU–low support condition comprised 25 girls and 15 boys (*n* = 40; *M*_age_ = 17.12, *SD* = 0.99), the private ASMU–high support condition comprised 20 girls and 19 boys (*n* = 39; *M*_age_ = 16.80, *SD* = 1.11), the public ASMU–low support condition comprised 23 girls and 16 boys (*n* = 39; *M*_age_ = 17.13, *SD* = 1.02), and the public ASMU–high support condition comprised 28 girls and 12 boys (*n* = 40; *M*_age_ = 17.31, *SD* = 0.96). To examine whether the four experimental groups were comparable in age and gender, we conducted a one-way ANOVA and a chi-square test. The ANOVA indicated no significant difference in age across the four groups, *F*(3, 154) = 1.70, *p* = 0.169. Similarly, the chi-square test indicated no significant difference in gender distribution across the four groups, χ^2^(3, *N* = 158) = 3.01, *p* = 0.390. Thus, the four experimental conditions did not differ significantly in age or gender.

Participants were included if they possessed at least one active social media account with a usage record in the past month and provided informed consent (guardian consent was required for minors). Exclusion criteria comprised a diagnosis of a mental disorder or receipt of psychotherapy within the past 6 months, as well as social media usage frequency below once per month (extreme low users). All exclusion criteria were assessed through participants’ self-report, as no clinical diagnostic records or external verification were available to the research team. Both studies employed a convenience sampling approach by posting advertisements on social media platforms to recruit participants, who received a monetary reward upon completion of the study.

### 3.2. Procedure

The experiment employed a 2 (active social media use type: public vs. private) × 2 (perceived online social support: high vs. low) between-subjects design. Participants were randomly assigned with equal probability to one of the four experimental conditions using the random assignment function of the survey platform Wen Juan Xing (Enterprise Standard Edition). The high versus low perceived online social support manipulation was delivered through scenario-based materials: participants in the high-support condition read a scenario in which the protagonist received supportive comments and explicit social validation, whereas those in the low-support condition read a scenario in which the protagonist received neutral or indifferent responses. Assignment to the high- or low-support scenario therefore determined each participant’s condition; no median split or pre-existing difference in perceived support was used. Each scenario was presented as a simulated social media interface (WeChat Moments for the public condition; a WeChat one-on-one conversation for the private condition). After reading the scenario, participants completed the manipulation-check item and the well-being measure.

### 3.3. Design and Measures

Online social support was manipulated using the scenario materials. In the public ASMU condition, participants in the high online social support group viewed a WeChat Moments image with a high number of likes and comments, while those in the low online social support group viewed an image with few likes and comments. In the private ASMU condition, participants either read a WeChat conversation with detailed and lengthy responses (high online social support group) or a conversation with brief and superficial responses (low online social support group).

To check the manipulation, each participant responded to the item, “The person who posted on Moments/initiated the conversation can perceive social support from the Moments/conversation” on a five-point Likert scale from 1 = *strongly disagree* to 5 = *strongly agree*. Next, well-being was assessed using the single-item measure of well-being developed and validated by Cheung and Lucas (2014), namely, “Now, how would you assess the satisfaction of the person who posted on Moments/initiated the conversation?”. Participants rated their responses on a five-point Likert scale from 1 = *very dissatisfied* to 5 = *very satisfied*.

### 3.4. Statistical Analyses

Data were analyzed using SPSS (Version 25; IBM Corp., Armonk, NY, USA). To verify the manipulation, a one-way analysis of variance (ANOVA) was conducted on the manipulation-check item, comparing the high- and low-support groups. The primary hypothesis was tested with a 2 (active social media use type) × 2 (perceived online social support) between-subjects ANOVA with well-being as the dependent variable. Significant interaction effects were followed up with simple-effects analyses (pairwise comparisons at each level of perceived online social support). Effect sizes are reported as partial *η*^2^.

### 3.5. Results

The results show that the manipulation check was indeed successful. Participants in the high online social support group (*M* = 3.53, *SD* = 0.89) reported higher perceived online social support than participants in the low online social support group (*M* = 2.50, *SD* = 0.77), *t* (156) = 7.76, *p* < 0.01. Moreover, independent t-test analysis indicated a significant main effect of ASMU type on well-being, *t* (156) = 5.94, *p* < 0.01. Specifically, participants in the public ASMU condition (*M* = 3.71, *SD* = 0.97) reported higher well-being than participants in the private ASMU condition (*M* = 2.83, *SD* = 0.92).

**Figure 3:**
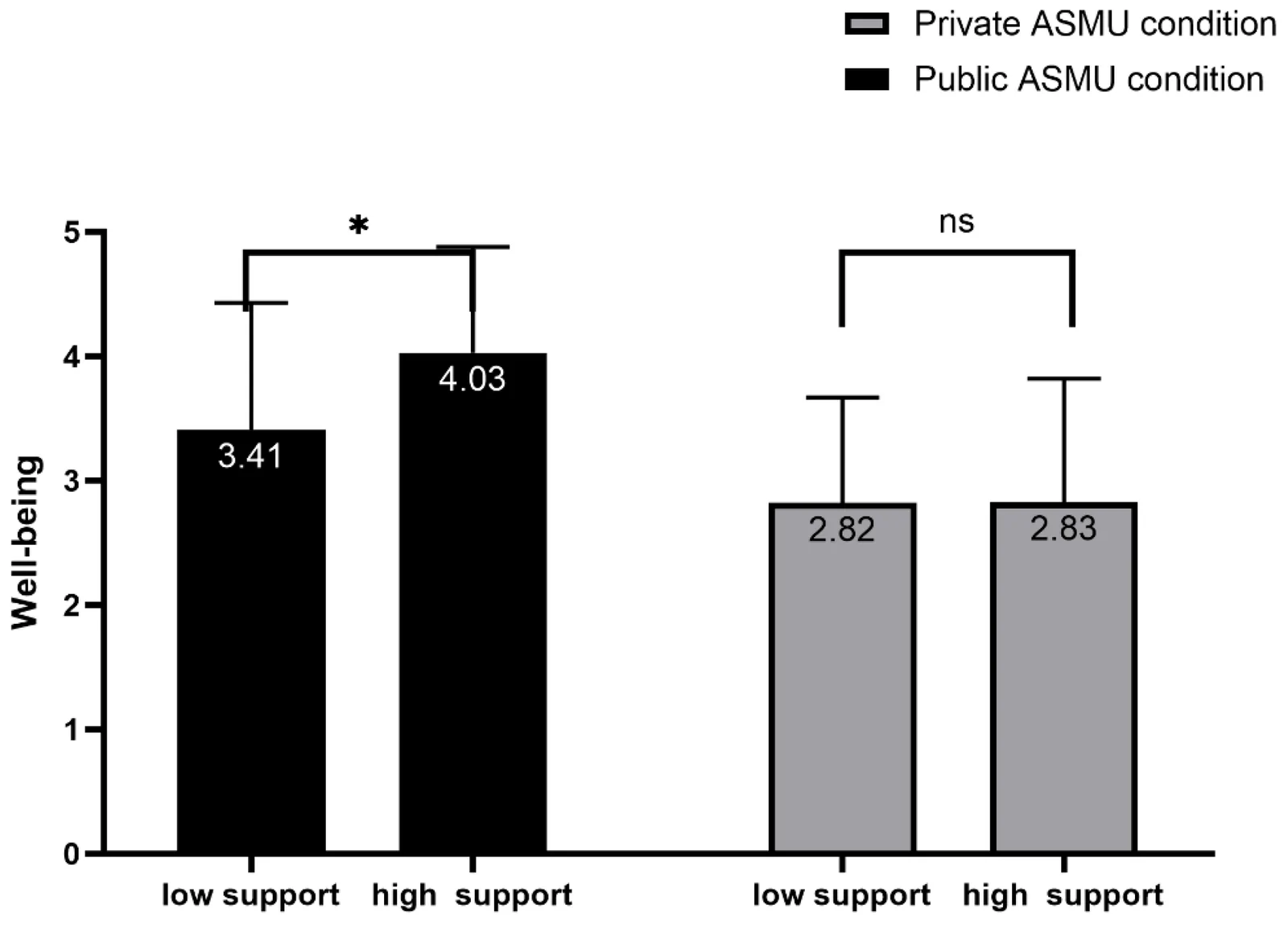
Interaction effect of public vs. private ASMU and perceived online social support on well-being. Note. * *p* < 0.05.

The ANOVA analysis revealed a significant interaction effect for ASMU type and perceived online social support on well-being, *F* (1, 154) = 6.42, *p* < 0.05 (See Figure 3). Consistent with the previous hypothesis 3, in the public ASMU condition, participants in the low online social support group (*M* = 3.41, *SD* = 1.02) were less likely to report well-being than those in the high online social support group (*M* = 4.03, *SD* = 0.81), *t* (76) = −3.20, *p* < 0.01. This indicates that perceived online social support moderates the relationship between public ASMU and well-being. Therefore, H3 is strongly supported. However, in the private ASMU condition, there was no difference in well-being between the high online social support group (*M* = 2.83, *SD* = 0.85) and the low online social support group (*M* = 2.82, *SD* = 0.99), *t* (78) = 0.06, *p* > 0.05. This indicates that perceived online social support did not moderate the relationship between private ASMU and well-being. Therefore, H4 was not supported.

### 3.6. Discussion

The findings of this experimental study provide further support for the results obtained in Study 1. Consistent with the findings of Study 1, Study 2 revealed that public ASMU had a stronger positive effect on well-being than private ASMU. This suggests that publicly visible interactions on social media platforms, such as posting status updates or sharing content, may serve as a more potent source of psychological benefits than private interactions, which often occur in closed, dyadic settings (Valkenburg et al., 2022). We note, however, that the moderation effect was modest in size, and that Study 2 assessed well-being with a single item framed as a third-person judgment of the fictional vignette character rather than the participant’s own well-being; the experimental support, although consistent, should therefore be interpreted with appropriate caution.

Study 2 also demonstrated the moderating role of perceived online social support in the relationship between public ASMU and well-being. Specifically, participants exposed to high levels of social feedback (i.e., numerous likes and comments) experienced greater subjective well-being than those in the low perceived online social support condition. This finding accords with the social enhancement hypothesis (Valkenburg & Peter, 2007), which posits that the positive outcomes of social media use are contingent upon the quality of online interactions (Valkenburg & Peter, 2007). In public ASMU, the visibility of supportive responses likely amplifies feelings of social connectedness and validation, thereby enhancing users’ well-being (Westmaas et al., 2020). In contrast, perceived online support did not moderate the relationship between private ASMU and well-being. One possible explanation for this is that private ASMU, such as chatting via messaging apps, involves more familiar, reciprocal, and routine exchanges that are less sensitive to variations in perceived support over a short time frame (e.g., a scenario-based experiment). Unlike public ASMU, where the tone and number of feedback are more salient and evaluative, private conversations may be perceived as inherently supportive, thus reducing the variability needed for a significant moderation effect to emerge (Valkenburg et al., 2022).

Despite the strengths of the experimental design, including random assignment and successful manipulation checks, certain limitations should be acknowledged. First, the study used a single type of scenario for each ASMU condition, but this may not fully capture the complexity and diversity of public and private ASMU in real-life contexts. For instance, private ASMU can include emotionally rich interactions such as peer disclosure or intimate conversations, which were not modeled in this study. Similarly, public ASMU may vary in tone, content type, and audience scope, all of which could alter its effect on well-being (Valkenburg et al., 2022). Second, although this study moves beyond correlational designs and provides initial causal evidence, the use of hypothetical scenarios may limit its ecological validity. Participants’ responses may not have fully reflected their actual emotional experiences when engaging in real-time online interactions. Therefore, future studies could benefit from incorporating ecological momentary assessment (EMA) or longitudinal diary designs to capture naturalistic ASMU behaviors and their dynamic associations with well-being (Shiffman, 2009). A further limitation specific to Study 2 is that well-being was measured with a single item asking participants to rate the satisfaction of the fictional person depicted in the vignette rather than their own well-being. This third-person framing assesses a social judgment about another person instead of the participant’s own well-being, which constrains the construct validity of direct comparisons with the self-reported well-being measure used in Study 1.

## 4. General Discussion

In modern society, social media have become an integral part of people’s lives. In this study, we explored the relationship between different types of ASMU and well-being, as well as the moderating role of perceived online social support, through Study 1 (a cross-sectional design) and Study 2 (a scenario-based experiment). Specifically, we examined how public and private ASMU affect individuals’ well-being, and how perceived social support influences these effects in both contexts.

### 4.1. Theoretical Implications

This study offers several important theoretical contributions. First, prior studies on the relationship between ASMU and well-being have yielded mixed findings, with some studies reporting positive effects (e.g., Verduyn et al., 2017), while others suggest no effects, or even negative effects (e.g., Valkenburg et al., 2022). One potential reason for these inconsistencies is the conceptualization of ASMU as a unitary construct, without accounting for the diversity of its forms. Addressing this gap, the present study is the first to distinguish between public ASMU (e.g., posting status updates, sharing content with a broad audience) and private ASMU (e.g., direct messaging, engaging in closed-group discussions) and systematically investigate their respective associations with well-being. The findings of both studies consistently indicate that public ASMU exerts a more positive effect on well-being than private ASMU. One plausible explanation for this is that public ASMU allows individuals to express identity-related content in a way that is more likely to garner feedback and recognition from a wider audience, thereby reinforcing their self-esteem and fostering a more positive self-concept (Marengo et al., 2021). In this sense, public ASMU may serve as a form of self-affirmation and social validation, which are key mechanisms known to promote psychological well-being (Valkenburg et al., 2022; James et al., 2022). By contrast, private ASMU is more likely to involve smaller, less visible interactions, and thus may not evoke the same level of positive reinforcement (Frison & Eggermont, 2020). Although private social media use can still foster a sense of connection, the limited scope of its interactions (e.g., one-on-one conversations or small groups) may reduce its effect on well-being compared to the more expansive reach of public posts. In summary, these findings highlight the importance of differentiating between types of ASMU when investigating their psychological consequences. Treating ASMU as a homogeneous construct may obscure meaningful differences in how various forms of engagement affect users’ well-being. Our results suggest that public ASMU, by enabling identity expression, social recognition, and broader interaction, may be more effective in enhancing subjective well-being compared to more restricted, private forms of use. These results theoretically advance prior media effects frameworks by differentiating the psychological impacts of public versus private social media features.

Second, our study revealed that online social support moderates the effect of public ASMU on well-being. Specifically, in the public ASMU condition, individuals who perceived high levels of online social support reported significantly greater well-being than those who perceived low levels of support. This indicates that public ASMU does not always lead to positive outcomes. This finding underscores the fact that the psychological benefits of public ASMU are not uniform but are instead contingent on the social context in which such engagement occurs. When users perceive their online audience as being supportive, public interactions (e.g., sharing personal updates) may yield affirming feedback (e.g., likes, encouraging comments), which in turn can enhance feelings of belonging, psychological safety, and self-worth (Valkenburg et al., 2022; Marengo et al., 2021). Conversely, when perceived support is low, such public exposure may result in social indifference or even rejection, potentially diminishing or reversing the positive outcomes. This interaction effect agrees with Social Support Buffering Theory (Cohen & Wills, 1985; Sarason, 2013), which posits that perceived support acts as a buffer that mitigates the negative psychological effects of stress and enhances the benefits of positive experiences. In the context of social media, a high degree of perceived online support may amplify the emotional gains associated with public self-disclosure and identity expression, thus enhancing the user’s well-being (Wright, 2016). This is particularly relevant given that public ASMU often involves some degree of social risk, as individuals expose their personal content to a broad and sometimes unpredictable audience. When that audience is perceived as supportive, the potential social risk is mitigated, and the rewards, such as increased social validation and connectedness, are maximized.

Third, in the private ASMU condition, different levels of perceived online social support did not significantly affect individuals’ well-being outcomes. This suggests that, unlike public ASMU, the psychological impact of private ASMU is less dependent on users’ broader perceptions of online support. One plausible explanation for this is that private ASMU typically occurs within more stable and familiar interpersonal contexts, such as interactions with close friends, romantic partners, or family members (Cole et al., 2017), where the availability of support is more predictable and internally regulated by the relationship itself. As Valkenburg et al. (2022) note, private interactions tend to be shaped by existing relational histories and are characterized by trust, intimacy, and habitual communication. In such contexts, support is often expected rather than actively evaluated, making moment-to-moment perceptions of broader online support less salient. This null result for private ASMU is consistent with a partial disconfirmation of H1, which had predicted a negative effect of private ASMU on well-being.

In sum, the present study extends our theoretical understanding by emphasizing the importance of differentiating between types of ASMU and by situating social media use within a more interactive and context-sensitive framework. Viewing ASMU as a heterogeneous set of behaviors rather than a single, uniform construct allows for a more accurate and nuanced interpretation of its psychological consequences.

### 4.2. Practical Implications

The findings of this study also have important practical implications for social media platforms, educators, mental health practitioners, and policymakers. First, our results suggest that public ASMU can be beneficial to users’ well-being, but this is primarily the case when it is accompanied by high levels of perceived online support. Therefore, digital platforms should be designed to foster more meaningful, affirming, and supportive interactions among users. Features that promote constructive engagement, such as the ability to highlight positive comments, facilitating peer recognition, and enabling feedback moderation, may help cultivate an environment in which public sharing leads to greater psychological benefits.

Second, researchers and mental health practitioners should be cautious in assuming that social media use has uniformly predictable benefits and should instead recognize that not all users experience the same level of support or benefit equally from similar engagement patterns. Although public ASMU can offer psychological benefits by providing opportunities for self-expression, identity affirmation, and social validation, these positive effects are not guaranteed across all user groups or contexts. As demonstrated by our findings, the psychological outcomes of social media engagement are highly contingent upon individual differences in perceived online social support. For some users, particularly those who feel embedded in a responsive and affirming online network, public sharing can lead to increased well-being through mechanisms such as increased self-worth, a sense of connectedness, and social reinforcement. However, for those who perceive low levels of support or encounter online environments marked by indifference, criticism, or exclusion, the same behaviors may yield neutral or even adverse emotional consequences.

Educationally, our findings offer concrete lessons for digital literacy and socio-emotional learning. Because the well-being benefit of public ASMU depends on perceived online support, schools and youth workers can help adolescents use public versus private channels more deliberately—fostering affirming networks, recognizing when public sharing is driven by self-presentation pressure rather than genuine connection, and turning to supportive one-on-one interactions when socially vulnerable. This is especially relevant for adolescents who perceive low online support.

### 4.3. Limitations and Directions for Future Research

Although this study contributes to a better understanding of the relationships between ASMU and well-being, it has several limitations that should be noted. First, Study 2 relied on a single scenario in a scenario-based, short-term experiment, which limits ecological validity and prevented examination of long-term effects; future work should vary scenarios and adopt longitudinal designs. Second, both samples were Chinese adolescents using WeChat exclusively, and Study 1 was strongly gender-imbalanced (≈76% female); caution is therefore warranted in generalizing to other platforms (e.g., Instagram, TikTok) or male-skewed populations, and cross-cultural comparisons are needed. Third, the assessment of ASMU and well-being was narrow. Public and private ASMU were each measured with only two frequency-based items that miss engagement depth (e.g., emotional investment, self-disclosure), and because public posts are curated to appear less offensive and more acceptable, the public-use measure may partly reflect positivity management rather than raw social activity. Relatedly, Study 2 assessed well-being with a single third-person item, limiting construct validity against Study 1’s self-report measure; future research should adopt comprehensive, multi-dimensional scales. Fourth, the design constrains conclusions about the direction and functional form of the ASMU–well-being link: Study 1’s cross-section leaves it open to reverse causation and self-selection (better-adjusted adolescents may simply use public ASMU more), and all models assumed linear associations without testing curvilinear effects, so possible non-linear patterns remain unexamined; longitudinal or panel designs testing non-linear forms are needed.

## 5. Conclusions

This study contributes to a more nuanced understanding of how different forms of active social media use (ASMU) relate to users’ well-being by distinguishing between public and private engagement. Through two studies, it revealed that (1) public ASMU is more strongly positively associated with well-being than private ASMU. (2) Perceived online social support moderates the relationship between public ASMU and well-being. Specifically, individuals who reported higher levels of perceived support experienced greater well-being following public ASMU, whereas those who reported lower perceived support derived less benefit. (3) Perceived online social support does not significantly moderate the relationship between private ASMU and well-being.

## Figures and Tables

**Table 1:** Means, standard deviations, and Pearson product-moment correlations of the study 1 variables.

	*M*	*SD*	1	2	3	4	5
1 Public ASMU	2.87	0.85	-				
2 Private ASMU	3.30	0.86	0.48 **	-			
3 Perceived online social support	2.75	0.77	0.20 **	0.30 **	-		
4 Well-being	3.04	0.79	0.08 **	−0.02	0.34 **	-	
5 Gender	1.76	0.43	0.08 *	0.12 **	0.09 *	−0.19 **	-

Note. * *p* < 0.05, ** *p* < 0.01. Gender was dummy-coded, such that *male* = 1 and *female* = 2.

**Table 2:** Moderating effects of perceived online social support in the relationships linking ASMU to well-being.

Predictors	Well-Being		
	β	*t*	95%CI
public ASMU	0.15	3.91 **	[0.04, 0.27]
private ASMU	−0.01	−0.25	[−0.13, 0.09]
perceived online social support	0.21	5.74 **	[0.12, 0.31]
public ASMU* perceived online social support	0.06	2.18 *	[0.01, 0.10]
private ASMU* perceived online social support	−0.01	−0.39	[−0.05, 0.03]
*R* ^2^	0.22		
*F*	19.04 **		

Note. * *p* < 0.05, ** *p* < 0.01.

## Data Availability

Data can be made available on reasonable request from the authors.
